# Academic writing challenges and supports for early-stage Chinese postgraduates: A mixed-methods study on teaching-research integration, supervisor engagement, and self-efficacy

**DOI:** 10.1371/journal.pone.0317470

**Published:** 2025-02-14

**Authors:** HuanHuan Zhang, XiaoShu Xu, Vivian Ngan-Lin Lei, Wilson Cheong Hin Hong, Weng Jie

**Affiliations:** 1 Faculty of Applied Sciences, Macao Polytechnic University, Macao, China; 2 School of Foreign Studies, Wenzhou University, Wenzhou, Zhejiang, China; 3 Centre for Portuguese Studies, Macao Polytechnic University, Macao, China; 4 Centre for Education Quality Management, Macao University of Tourism, Macao, China; Ahvaz Jundishapur University: Ahvaz Jondishapour University of Medical Sciences, ISLAMIC REPUBLIC OF IRAN

## Abstract

Enhancing academic publication outcomes and writing skills in postgraduates is a key goal for higher education institutions, ideally through early intervention. This study examines the challenges and support needs of early-stage Chinese postgraduates in academic writing, as well as the factors influencing their publication outcomes and writing self-efficacy. Using snowball sampling, 73 postgraduates were surveyed through questionnaires and reflective journals. Thematic analysis and linear regression were applied to analyze the data. Findings reveal primary challenges in literature review, research methods, data analysis, and topic selection. Key support needs include supervisor guidance, knowledge resources, tools, and self-assessment. Results indicate that the year of study, meeting frequency, supervisor support, teaching-research nexus, and self-efficacy do not significantly impact publication outcomes. However, supervisor support and the quality of the teaching-research nexus positively influence self-efficacy. These findings highlight the importance of providing targeted support and fostering intrinsic motivation to enhance the writing skills and self-efficacy of early-stage postgraduate students.

## Introduction

Postgraduate students’ academic writing requires a critical set of skills, including conducting research, analyzing data, and effectively communicating complex ideas [1, 2]. However, postgraduate students face numerous challenges in academic writing, such as concerns about plagiarism and originality, language barriers for non-native English speakers, vague language use, grammatical errors, and a lack of coherence in writing [3]. These issues threaten the integrity and clarity of their work and impact their overall academic performance. Therefore, addressing these challenges through targeted educational support is essential for enhancing the academic writing skills of postgraduate students [2, 3].

The integration of teaching and research is gaining attention in the global higher education landscape, recognized as pivotal to fostering academic excellence and innovation, particularly in postgraduate education. This integration transcends the mere incorporation of research activities into teaching; it cultivates an academic environment where teaching and research are interdependent and mutually beneficial, enhancing the quality and effectiveness of postgraduate education. A significant indicator of the effectiveness of this integration is the academic achievements of learners, making it essential to explore the impact of the teaching-research nexus on learners’ academic outcomes. Teaching and research activities span multiple dimensions, including traditional classroom settings and faculty engagement. The depth and quality of interaction between postgraduate students and their academic mentors are critical in shaping academic outcomes, highlighting the substantial influence of faculty in guiding students’ research trajectories, academic inquiries, and nurturing a supportive educational atmosphere [4]. In the Chinese context, the extent to which different institutional structures and levels of faculty involvement affect students’ research skills development and overall academic achievements merits further investigation [5, 6].

Moreover, in postgraduate education, academic writing self-efficacy—the confidence students have in their ability to engage in scholarly writing—is widely acknowledged as an important factor affecting academic writing performance and overall academic achievement [7]. Students with higher self-efficacy are more likely to actively participate in academic activities, demonstrate stronger problem-solving abilities, and exhibit resilience in the face of academic challenges. This self-efficacy significantly impacts the publication of students’ academic outcomes and is essential in the academic writing process. However, cultivating academic writing self-efficacy is complex, influenced by various factors such as faculty guidance [8], academic writing retreats [9], and academic writing resources like Wikis [10]. While the teaching-research nexus is a hot topic in educational discourse and a strategy for improving educational quality, few have explored it as a factor affecting students’ academic self-efficacy. Therefore, studying it as an influencing factor would broaden the scope of factors affecting academic writing self-efficacy and enhance the practical significance of the teaching-research relationship.

This study aims to fill a critical gap in the existing literature by exploring the collective impact of the teaching-research nexus, supervisor support, and academic writing self-efficacy on the research outcomes of early-stage postgraduate students in China. It also investigates the influence of the teaching-research nexus and faculty engagement on academic writing self-efficacy. Addressing this gap is crucial for improving these students’ academic quality and research productivity, thereby helping elevate the standards of higher education in China to an international level. While previous studies have typically examined these factors in isolation, mainly in the European context, there is a lack of integrated research focusing on their combined effects within the unique framework of Chinese higher education. This investigation seeks to fill this void, providing insights into how these interconnected factors influence the research outcomes of early-stage postgraduate students in China.

## Literature review

### Challenges of postgraduates’ academic writing publication

Early-stage postgraduate students face numerous academic writing challenges. Qasem and Zayid [11] highlighted obstacles such as limited proficiency in academic English, which undermines their writing effectiveness. This issue is especially pronounced in regions like Pakistan, where English is the primary language for instruction and academic publishing [3, 12]. Despite high expectations for clarity and logic, many postgraduates struggle with academic writing in English, emphasizing the need for better support and instructional resources.

Beyond language barriers, academic writing is complicated by issues of originality and the risk of plagiarism. In Turkey, for instance, postgraduate theses exhibit high similarity indices and alarming rates of plagiarism, with an average similarity index of 25.10 and 34.5% of reviewed papers containing plagiarized content [2].

Additionally, Qasem and Zayid [11] emphasized difficulties in selecting research topics, mastering methodologies, and finding appropriate literature, indicating a need for better foundational research skills. Further challenges include waning research interest, superficial subject understanding, insufficient supervisor guidance, and time management concerns.

While these obstacles are significant, targeted guidance and supportive educational practices like Project-Based Learning (PBL) [1] and peer feedback [12] can improve writing skills and academic outcomes.

### Supports needed for postgraduates’ academic writing publication

Enhancing postgraduate academic writing skills requires a comprehensive approach addressing skill development, support networks, and structured interventions. For non-English speaking students, targeted support is essential to master academic vocabulary, construct coherent arguments, and align writing with academic expectations [13].

Support networks, particularly guidance from supervisors on content and writing skills, are crucial. Feedback on language use and adherence to deadlines underscores the importance of a supportive supervisory relationship. Institutional support services, including writing workshops, peer support groups, proofreading services, and ICT tools, are also essential [13].

Structured interventions should include pedagogically driven writing support integrated into the curriculum and delivered by experts. Supervisors need training in pedagogical strategies for effective writing support. Additionally, targeted peripheral support services like academic writing classes, online tutorials, and administrative guidance should be systematically provided [13].

Early-stage postgraduate students face numerous hurdles, including limited English proficiency, difficulty selecting research topics, mastering methodologies, and sourcing literature [11]. Waning research interest, superficial subject understanding, insufficient supervisor guidance, and time management concerns highlight the need for holistic support measures. Specialized language support, comprehensive research methodology training, stronger mentorship, and time management strategies are crucial for bolstering postgraduate academic achievements.

### Factors that impact postgraduates’ academic writing publication

Multiple factors influence academic achievement. Hawari et al. [14] identified the coherence and quality of academic papers as critical, suggesting detailed feedback and strategies for text structure and summarization can enhance writing quality. Shen et al. [15] indicated the syntactic complexity, widely acknowledged as a key predictor of writing quality, has gained increasing attention in the realm of academic writing. Sun et al. [16] found a significant correlation between self-efficacy in English writing and writing achievement, while Teng and Wang [17] showed academic self-efficacy beliefs predict performance in EFL academic writing. Cultivating self-efficacy empowers students to regulate their writing process and persist through challenges [13].

Cultural and linguistic backgrounds significantly impact students’ ability to master academic English and effective writing styles [18]. students’ perceptions and attitudes towards academic writing, influenced by their experiences, play a pivotal role, with positive attitudes linked to successful writing outcomes [19]. Disciplinary demands require alignment with specific conventions and standards, facilitated by the teaching-research nexus [20, 21].

Interpersonal dynamics, particularly supervisor feedback quality and timing, significantly affect writing development. Informative feedback that challenges students cognitively and encourages metacognitive awareness leads to deeper understanding and improved text quality [22]. Support from peers and academic networks, along with a positive institutional environment, helps foster a supportive research and publication culture [14].

These factors highlight the complexity of postgraduate academic writing, emphasizing the need for a supportive ecosystem with clear models of good practice, discipline-specific guidance, institutional support, and engaged mentorship. This paper aims to fill a gap by examining the confluence of the teaching-research nexus, supervisor support, and academic writing self-efficacy on postgraduate research outcomes in China.

### Factors that impact postgraduates’ academic writing self-efficacy

Academic writing self-efficacy, defined as students’ confidence in their writing abilities, significantly influences their writing performance and academic achievements [23]. Chung et al. [8] emphasized that instructor guidance in writing, planning, and goal setting, coupled with self-reflection, enhances students’ self-efficacy and writing outcomes. Vincent et al. [9] demonstrated that writing retreats, which include goal-setting and time management practices, bolster doctoral candidates’ writing self-efficacy and self-regulatory abilities. Additionally, Rahimi and Fathi [10] found that Wiki-mediated collaborative writing positively impacts EFL students’ writing performance and self-efficacy.

Learning motivation and writing self-efficacy are closely related. Izawa et al. [24] found a correlation between self-efficacy and learning motivation, where perceived progress enhances learning drive and self-efficacy. Schunk and DiBenedetto [25] supported this, indicating self-efficacy influences motivation. Despite valuable insights, understanding academic writing self-efficacy requires considering a broader range of variables.

### Theoretical framework

#### Social support theory

The theoretical foundation of this study is underpinned by House and Kahn’s [26] Social Support Theory, which outlines four critical dimensions of support instrumental in influencing academic achievement: emotional, instrumental, informational, and appraisal assistance. Emotional assistance refers to the provision of care, empathy, and understanding that foster a sense of acceptance and concern for the individual. This support can originate from family members, friends, colleagues, or professionals and is often conveyed through listening, encouragement, and comfort.

Instrumental assistance pertains to the supply of tangible aid, such as tools, financial support, materials, or services, equipping individuals with the necessary resources to manage emergencies or fulfill day-to-day needs. Informational assistance involves offering advice, guidance, or knowledge that empowers individuals to navigate challenges more effectively. Appraisal assistance primarily relates to the provision of feedback, validation, and affirmation, helping individuals assess their capabilities and worth.

Guided by Social Support Theory, this study employs reflective diaries to explore early-stage postgraduate students’ perceptions of academic writing. This approach delves into the emotional support students require, such as care and encouragement, assessing the affective support necessary for their well-being. It also scrutinizes the informational support needed, focusing on knowledge, guidance, and advice. Additionally, the study explores the instrumental support required, highlighting the tools, material resources, and services necessary for academic writing. Lastly, it examines the evaluative support needed, particularly self-assessment and feedback from others.

By leveraging this theory, the study aims to elucidate the types of support early-stage postgraduate students require in academic writing, enabling educational institutions and educators to provide more targeted guidance.

#### Basic requirements for academic paper writing

This study’s theoretical framework is informed by Yang’s [27] delineation of the fundamental components of academic writing in Academic Writing Coursebook for Postgraduates, which are categorized into eight parts: topic selection, abstract and keywords, introduction, literature review, methodology, results, discussion, and conclusion. Based on this framework, the research analyzes the challenges early-stage postgraduate students encounter regarding the basic structure of academic papers.

Furthermore, Yang [27] in the same publication, identifies four linguistic characteristics that academic writing must satisfy: unity of meaning, coherence in logic, clarity of expression, and variety in syntactic structure. These characteristics are emphasized as prerequisites for ensuring the quality of academic papers. Accordingly, this study explores the linguistic challenges students face in the academic writing process, using these four attributes as the benchmark.

### The present study

To address the identified gaps in the literature, this study examines the interactive effects of the teaching-research nexus, supervisor support, and academic writing self-efficacy on the academic publication outcomes of early-stage postgraduate students. It also investigates the impact of the teaching-research nexus and supervisor support on students’ academic self-efficacy. Addressing this research gap could lead to more targeted and holistic interventions in postgraduate education. Thus, this study aims to answer four research questions:

1. What challenges do Chinese early-stage postgraduates encounter in academic writing?
2. What forms of support are essential for Chinese early-stage postgraduates to enhance their academic writing skills?
3. How do the teaching-research nexus, supervisor support, and academic writing self-efficacy affect the academic publication outcomes of Chinese early-stage postgraduates?
4. How do the teaching-research nexus and supervisor support influence the academic writing self-efficacy of Chinese early-stage postgraduates?

## Methodology

### Sample and sampling

Snowball sampling, a non-probability technique, is used to recruit postgraduate participants, particularly effective when a comprehensive population list is unavailable [28]. Initial subjects (seed participants) refer additional participants, creating a chain of referrals [29]. This method suits the diverse and dispersed postgraduate population across various disciplines and institutions in China, reaching a broad sample when comprehensive directories are limited [30]. Using WeChat leverages existing social networks, efficiently spreading the survey link among academic communities.

The study involved 73 postgraduate participants from various academic disciplines across China, including but not limited to Science and Engineering, Humanities and Social Sciences, Medicine, and Business. The age of participants ranged from 22 to 40 years, with a median age of 26. The gender distribution was approximately 63% (46) female and 37% (27) male, aligning with general postgraduate demographics in Chinese universities. The years of postgraduate experience varied among participants, with 95% being first-year students and 5% in their second year of postgraduate studies.

### Research design

This study investigates the factors influencing postgraduate academic writing, publication outcomes, and academic writing self-efficacy in China. A mixed-methods design was adopted to leverage the complementary strengths of quantitative and qualitative approaches. This methodological framework facilitates the collection of broad, generalizable data through surveys while also capturing rich, detailed insights through reflective diaries, enabling a comprehensive understanding of the research topic [31]. To achieve these objectives, the study utilized reflective diaries and three specialized questionnaires as primary data collection tools, focusing on postgraduates’ experiences, challenges, and support needs.

The quantitative component involved the administration of three distinct questionnaires. The Teaching-Research Nexus Questionnaire, adapted from Devesh and Nanjundaswamy [32], was expanded to include 29 items covering seven dimensions: supervisor support, learning environment, skills development, research component, collaboration and publication, teaching and learning quality, and student motivation. The adaptation process involved rigorous exploratory factor analysis to ensure validity and reliability.

The Postgraduate Research Outcomes Questionnaire, developed specifically for this study, gathered data on research outcomes such as publication rates and academic networking opportunities. It included a mix of Likert-scale items, multiple-choice questions, and open-ended responses to provide a comprehensive view of postgraduate research achievements.

The Situated Academic Writing Self-Efficacy Scale (SAWSES), with 16 Likert-type items, assessed academic writing self-efficacy. Although originally identified as two latent constructs, for this study, it was treated as one overarching construct based on the literature, ensuring its relevance in the Chinese postgraduate context. Additionally, an ad hoc 20-question demographic questionnaire collected basic information and assessed factors influencing academic writing self-efficacy using a 5-point Likert scale.

The qualitative component was implemented through reflective diaries, where participants documented their academic writing challenges and the support mechanisms they found beneficial. This method provided rich, personal insights into the struggles and requirements of postgraduates, complementing the quantitative data with nuanced understanding.

### Instruments

Participants documented their academic writing challenges and the support mechanisms they found beneficial through reflective diaries. This method offers valuable personal insights into the struggles and requirements of postgraduates, enriching the data collected through other means and providing a nuanced understanding of their academic writing experiences.

A comprehensive questionnaire that contained four major sections (see S1 Appendix), demographic information, teaching-research nexus [32], the Situated Academic Writing Self-Efficacy Scale (SAWSES) [33] and postgraduate research outcomes, respectively. The data’ s suitability for factor analysis was confirmed by the Kaiser-Meyer-Olkin measure and Bartlett’s Test (KMO = 0.828; p < 0.001), indicating strong validity for factor analysis.

As for the teaching-research nexus, the original 21-item instrument, which spanned six dimensions—Research Led, Research Oriented, Research Based, Research Tutored, Quality (Teaching and Learning), and Motivation—was thoughtfully expanded to 29 items. This augmentation incorporated eight items about mentorship support, informed by robust theoretical frameworks such as Mentorship Theory, Social Support Theory, and Self-Determination Theory (SDT). Subsequently, the instrument was meticulously refined using exploratory factor analysis (EFA), ensuring that the modifications were grounded in both statistical rigor and theoretical integrity. By employing Promax rotation, the study facilitated intercorrelation among factors, acknowledging the complex interplay of educational constructs. Item selection prioritized items with loadings exceeding .04, ensuring theoretical congruence with the conceptual model. This rigorous refinement process culminated in a 26-item questionnaire structured around seven dimensions—supervisor support, learning environment, skills development, research component, collaboration and publication, teaching and learning quality, and student motivation (see Table 1).

**Table 1:** The teaching-research nexus’ structure matrix.

		Component						
		1	2	3	4	5	6	
Supervisor support	Q7	0.864						
	Q8	0.786						
	Q9	0.870						
	Q10	0.808						
	Q11	0.826						
	Q12	0.754						
	Q13	0.839						
	Q14	0.759						
Learning environment	Q15		0.794					Removed
	Q16			0.488				
	Q17			0.822				
	Q18				0.747			Removed
	Q19			0.776	0.431		0.442	
Skills development	Q20				0.713			Removed
	Q21			0.534				
	Q22			0.797				
	Q23			0.824				
Research component	Q24				0.845			
	Q25				0.578			
	Q26				0.425			
Collaboration and publication	Q27					0.779		
	Q28					0.655		
	Q29					0.441		
Teaching and learning quality	Q30		0.519					
	Q31		0.558					
	Q32		0.789					
Student motivation	Q33						0.791	
	Q34						0.554	
	Q35						0.539	

*Note*: Refer to S1 Appendix for the corresponding questions

Rotation Method: Promax with Kaiser Normalization.

As for the SAWSES, a recently developed measurement tool, is used to assess academic writing self-efficacy in this study. The SAWSES includes 16 Likert-type items identified as two latent constructs (see Table 2), but for this study, it is treated as one overarching construct based on existing literature. The SAWSES is specifically designed to measure academic writing self-efficacy, which is critical for the academic success of postgraduate students. This makes it highly relevant for assessing the self-efficacy of first-year postgraduate students in a Chinese university setting, bridging the gap between Western and Eastern educational evaluations and enriching our understanding of academic writing self-efficacy across diverse populations.

**Table 2:** SAWSES’ structure matrix.

	Component	
	1	2
Q36	0.729	
Q37	0.758	
Q38	0.729	
Q39	0.838	
Q40	0.702	
Q41	0.813	
Q42	0.811	
Q43	0.660	
Q44	0.607	
Q45		0.770
Q46		0.736
Q47		0.834
Q48		0.867
Q49		0.896
Q50		0.770
Q51		0.822

*Note*: Refer to S1 Appendix for the corresponding questions

Rotation Method: Promax with Kaiser Normalization.

As for the postgraduate research outcomes was specifically developed for this study to collect data on various research outcomes, including publication rates and academic networking opportunities. Its purpose is to understand the influence of institutional factors on postgraduate research achievements. The part features multiple-choice questions, and open-ended responses, providing a comprehensive picture of postgraduate research accomplishments and the role of the academic environment in these outcomes.

### Data collection

Data for this study were collected using a multifaceted approach that included a comprehensive questionnaire that contained three major sections, and a reflective diary component, thoroughly capturing the academic writing experiences of early-stage Chinese postgraduates. The data collection for this study began on February 29, 2024, and concluded on April 12, 2024. participants were instructed in a classroom setting to begin their reflective diaries, focusing on their academic writing challenges and the effectiveness of various support mechanisms. Digital notebooks were recommended for their convenience and ability to capture immediate reflections, providing rich, personal insights into the academic hurdles and support strategies from the students’ perspectives.

The comprehensive questionnaire was distributed through the Wenjuanxing platform. This platform targeted a wide audience of postgraduate students across diverse Chinese higher education institutions via WeChat, ensuring broad participation while maintaining the anonymity and confidentiality of responses.

### Data analysis

Linear regression analysis was conducted to explore the relationships between the proposed factors and two key academic outcomes: academic writing publication success and self-efficacy in academic writing. The independent variables (IVs) included elements associated with supervisor support (SS) and dimensions of the teaching-research nexus, such as learning environment (LE), skill development (SD), research component (RC), teaching and learning quality (TL), motivation (MO), and collaboration and publication (CP). Each independent variable was entered individually into separate regression models to assess its unique influence on the dependent variables (DVs): academic writing publication outcomes and academic writing self-efficacy. This approach provided clear, interpretable results while ensuring that the analysis remained focused on specific, significant relationships.

For qualitative data, thematic analysis of reflective diaries was conducted following Braun and Clarke’s six-phase framework [34], which included familiarization with the data, generating initial codes, searching for themes, reviewing themes, defining themes, and writing up results. NVivo software was employed to facilitate the organization and coding of the diary entries, allowing for systematic identification of prevalent themes. The analysis focused on challenges commonly reported by participants and the types of support mechanisms sought, ensuring alignment with the study’s objectives. Themes that were most relevant to the research questions and had the strongest implications for academic writing practices were prioritized for discussion. Secondary themes are summarized briefly or provided in supplementary materials to maintain a focused narrative in the discussion.

### Ethics statement

This study strictly adheres to the guidelines of the Academic Committee of Wenzhou University in China, ensuring that ethical considerations are fully complied with. As participants were recruited from Wenzhou University, ethical approval was required. Emphasizing ethical standards is crucial for protecting the welfare and rights of participants. We submitted a detailed application to the Wenzhou University’s Institutional Review Board (IRB), covering the research objectives, methods, potential risks, and protective measures provided for participants. After a comprehensive review, Wenzhou University Review Board approved the study, approval number (IRB/IORG #202401) on 16 January 2024, acknowledging the ethical integrity of the study and the commitment to maintaining the dignity and confidentiality of participants.

### Informed consent

All participants in this study underwent a rigorous informed consent process to ensure adherence to ethical standards. Participants signed consent forms that detailed the scope of the study and their rights. These electronic consent documents are well-maintained and can be provided upon request during peer review or after publication. The study protocol is designed to ensure that all participants fully understand the research objectives, procedures, potential risks, and benefits before providing their consent. All participants in this study are adults and possess full decision-making capacity. The Wenzhou University Review Board confirms the ethical compliance of the study, including the oversight of the informed consent process.

## Results

### Results of the reflective diary

The reflective journal entries from students were transcribed and organized into sequentially numbered files within a Students’ Reflective Journal’ directory. Following Braun and Clarke’s [34] methodology, the first and second authors jointly undertook the coding process. Initially, the first author conducted an exploratory review of the text, generating a preliminary code list. This list was then refined through discussions between the two researchers, leading to the identification of emergent themes. Both authors separately performed a thematic analysis using these initial codes, a step critical for ensuring rigorous and impartial data interpretation.

The collaboration between the researchers resulted in an agreement rate exceeding 90% on the themes, codes, and referenced instances, indicating strong inter-coder reliability. Any differences were reconciled through detailed comparison and discussion, sometimes necessitating the redefinition of codes and the rearrangement of themes. A second round of coding was then conducted based on the adjusted themes, resulting in a definitive list of themes and codes, as illustrated in Tables 3 and 4.

**Table 3:** Principles for challenges encountered in academic writing.

Main themes	Subthemes	Example quotes
Selection of topics	Determination of the selected topic	Difficulty in Choosing a Topic: Unsure of how to select an appropriate research topic, or finding a direction worth exploring in depth within existing studies.
	Feasibility	Determining whether a topic is valuable requires considerable preliminary accumulation and attention to cutting-edge theories and technologies. It is challenging to identify a valuable and feasible topic.
	Novelty	The chosen topic for a thesis may lack innovation and not have a clear point of innovation.
	Valuability	Selecting a valuable and feasible research question is the primary challenge in academic paper writing.
Title	Formatting of the title	For example, the format of the title, abstract, references, etc., is incorrect.
Abstract and keywords	Formatting of the abstract and keywords	For example, the format of the title, abstract, references, etc., is incorrect.
	Writing of abstract and keywords	The abstract should offer a concise summary, and the keywords should be succinctly refined.
Introduction	Writing of introduction	My introduction section is not very clear on how to effectively introduce the problem.
Literature review	Citation	How to cite and explain ideas from the literature in your paper in a sensible way.
	Literature collection and collation	Literature search does not know how to effectively find and screen literature, the result is to find a lot of irrelevant information, or miss important literature, so that the article written is somewhat one-sided or inaccurate.
	Writing of literature review	For example, I know that literature review is to sort out the literature, but according to what logic to sort out? What is the general chain of logic? When writing, it is easy to become a stack of literature.
Methodology	Selection of research methods	Decide which research methodology to use to conduct the study and at the same time ensure the appropriateness and reliability of the methodology.
	Participants	Lack of reliable data sources or sample size.
	Data collection	Not sure where the data should be found.
	Data analysis	No analytical tools will be used in the collection and collation of data for analysis.
	Instrument	Cannot design questionnaires and interviews.
	Research design	Experiments can’t be designed without ideas.
Research question	Determination of the research question	The selection of topics is not innovative enough and is not very good at identifying appropriate research questions.
Research theory	Construct of the research theory	Due to lack of familiarity with the theoretical underpinnings of my field of study, I had difficulty in constructing a theoretical framework for my thesis.
Result	Writing of result	Difficulty in interpreting results
Discussion	Writing of discussion	My weak point was in the discussion section, not being very well organised or literature combed and not quite sure exactly how to present this section.
Conclusion	Writing of conclusion	It is difficult to write the conclusion part of the paper
Reference	Formatting of the reference	Citation and reference formatting errors
The overall of the paper	Writing of the overall paper	before the class, it was actually easy to see what parts of the paper consisted of by reading a few articles, but crucially it wasn’t clear what should be written when it came to each part specifically.
	the whole components of the paper	The brain doesn’t have the full structure of an essay and the ideas are confused.
	Creativity of the paper	There are also issues such as lack of innovation, where the content of the paper lacks novelty and fails to contribute meaningfully to existing research.
Language Expression	Difficulty in expressing language	My weakness is in written expression, and I struggle to put ideas into words effectively.
	Clarity	I also have some problems with my level of language expression. Despite the desire for language to be as concise as possible, there are often cumbersome and even verbose expressions.
	Coherence	The writing skills are not good, the lines do not flow well, and there is a hard succession from paragraph to paragraph.
	Formality	It is important to learn how to write to look academic and professional, less colloquial and more professional.
English language challenge	Difficulty reading English	In reading the English literature, it was also necessary to use the relevant translation software, resulting in a poorly documented framework for the thesis.
	Difficulty writing in English	In English paper writing, there is no complete system of better language because of the current accumulation of fewer utterances, slower writing, and the need to use tools.

**Table 4:** Principles for support needed in academic writing.

Main themes	Subthemes	Example quotes
Emotional assistance		If we feel stressed or anxious during the writing process, we can seek the school’s counselling services to help keep ourselves in a good state of mind.
Appraisal assistance	Others appraisal-assistance	Seek feedback and guidance: ask professors, classmates, or other professionals to review your essay and suggest revisions to improve your writing.
	Self appraisal-assistance	Think and practice a lot: think carefully about your argument before you write, and work on gathering material and ideas in advance.
Instrumental assistance	Instruments	In terms of data analysis, some data may require specific software to analyse, and guidance is needed on how to use the software, etc. About the summary of results analysis, there is a need for continued learning on the use of data analysis software.
	Materiel and resources	Use campus resources: many schools offer resources such as libraries, writing centres, study groups, etc., which are valuable learning resources.
	Service	Provide hands-on instruction in quantitative and qualitative research methods, as well as training in data analysis skills to help students conduct effective research analyses using different methods
Informational assistance	Guiding	Instruction in writing skills, literature organisation and reading tools, instruction in how to cite literature.
	Recommendation and advice	Instructors can provide writing guidance and revision suggestions for students’ papers to help me improve my writing and the quality of the paper
	Knowledge	Learning and mastering academic writing skills: read relevant academic writing guides or textbooks to learn how to make sound arguments, organise essay structures and improve presentation skills.

### Challenges encountered by postgraduate students in the academic writing process

**Fig 1:**
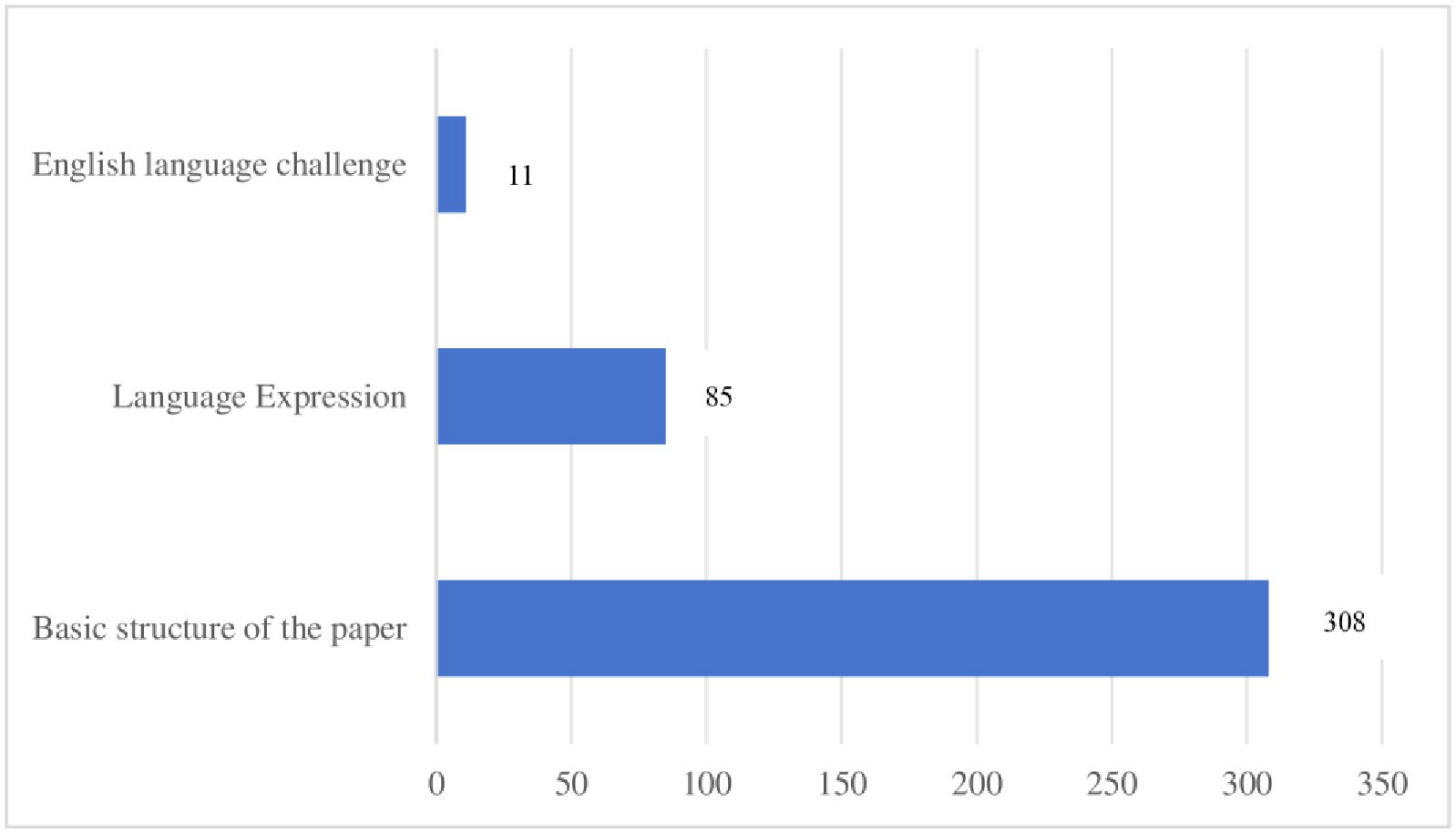
The challenges encountered by the students in academic writing. The figure provides an overview of the distribution of challenges encountered by students in academic writing, which are categorized into three main areas: the basic structure of the paper, language expression, and English language challenges.

For the challenges encountered by students in academic writing, reflective diaries from all participants were collected, resulting in a total of 404 items. These items were categorized into three main areas: 308 items related to the basic structure of the paper, 85 items concerning language expression, and 11 items addressing English language challenges (see Fig 1). These items were then organized into three independent data sets, as detailed in Tables 5 and 6.

**Table 5:** Challenges encountered in the basic structure of the paper.

Themes	R	Subthemes	R
Selection of topics	66 (21.4%)	Determination of the selected topic	34 (11.04%)
		Feasibility	9 (2.92%)
		Novelty	13 (4.22%)
		Valuability	10 (3.25%)
Title	1 (0.3%)	Formatting of the title	1 (0.3%)
Abstract and keywords	3 (0.97%)	Formatting of the abstract and keywords	1 (0.3%)
		Writing of abstract and keywords	2 (0.65%)
Introduction	1 (0.3%)	Writing of introduction	1 (0.3%)
Literature review	82 (26.62%)	Citation	8 (2.60%)
		Literature collection and collation	29 (9.42%)
		Writing of literature review	45 (14.61%)
Methodology	78 (25.32%)	Selection of research methods	16 (5.20%)
		Participants	4 (1.30%)
		Data collection	17 (5.52%)
		Data analysis	29 (9.42%)
		Instrument	2 (0.65%)
		Research design	10 (3.25%)
Research question	8 (2.59%)	Determination of the research question	8 (2.59%)
Research theory	7 (2.27%)	Construct of the research theory	7 (2.27%)
Result	3 (0.97%)	Writing of result	3 (0.97%)
Discussion	5 (1.62%)	Writing of discussion	5 (1.62%)
Conclusion	4 (1.30%)	Writing of conclusion	4 (1.30%)
Reference	3 (0.97%)	Formatting of the reference	3 (0.97%)
The overall of the paper	47 (15.25%)	Writing of the overall paper	24 (7.79%)
		the whole components of the paper	11 (3.57%)
		Creativity of the paper	12 (3.40%)
Total	308	Total	308

*Note*: R stands for the reflective diaries.

**Table 6:** Challenges encountered in the language expression and English usage.

Themes	R	Subthemes	R
language expression	85 (88.54%)	Difficulty in expressing language	15 (15.63%)
		Clarity	14 (14.58%)
		Coherence	23 (23.96%)
		Formality	33 (34.38%)
English language challenge	11 (11.46%)	Difficulty reading English	5(5.21%)
		Difficulty writing in English	6 (6.25%)
Total	96	Total	96

The analysis of the reflective diaries revealed several challenges students encountered in the basic structure of their academic papers. The most frequently mentioned issue was the literature review, with 82 items noted. Other significant challenges included methodology (78 items) and topic selection (66 items). The structure of the paper was also a major challenge, accounting for 15.25% of the total mentions.

In terms of subthemes, students frequently addressed components of the paper’s basic structure. The most prevalent challenge was writing the literature review, accounting for 14.61% of occurrences. This was followed by determining the selected topic (11.04%), literature collection and collation (9.42%), and data analysis (9.42%). Writing the overall paper was also significantly mentioned, making up about 7.79% of the total occurrences.

Table 6 details the challenges students faced in terms of language expression and English language usage. where students are more concerned with the problems of expressing themselves in the language (88.54%), compared to the difficult aspects of English language use (11.46%). Participants more referred to these issues including formality, coherence, difficulty in expressing language, and clarity, accounting for 34.38%, 23.96%, 15.63%, 14.58% of responses, respectively.

### Support needed by postgraduate students in the academic writing process

Table 7 presents the thematic analysis from the second reflective diaries, totaling 155 codes. In the analysis, students predominantly needed informational assistance such as guiding, knowledge recommendation, and advice, Specifically, guiding and knowledge were the most mentioned, which accounted for 21.94% and 21.29% of occurrences, respectively. Followed by the appraisal assistance (22.58%) and instrumental assistance (22.58%), In this, self-appraisal assistance (18.06%) and instrumental support (10.97%) were most needed by students. Lastly, emotional assistance was rarely mentioned, constituting only 1.94% of the entries.

**Table 7:** Support needed in academic writing.

Main themes	R	Subthemes	R
Emotional assistance	3 (1.94%)		3 (1.94%)
Appraisal assistance	35 (22.58%)	Self-appraisal assistance	28 (18.06%)
		Others-appraisal assistance	7 (4.52%)
Instrumental assistance	35 (22.58%)	Instruments	17 (10.97%)
		Materials and resources	8 (5.16%)
		Service	10 (6.45%)
Informational assistance	82 (52.90%)	Guiding	34 (21.94%)
		Knowledge	33 (21.29%)
		Recommendation and advice	15 (9.68%)
Total	155	Total	155

### Results of factors affecting the academic writing publication outcome

After the inspection of the Variance Inflation Factor (VIF), all IVs were confirmed to be below 5 [35], although some were marginally acceptable. Hence, all IVs were run with one model. The main effect of the regression model was not significant, F (10, 62) = .772, *p* = .655. Table 8 presents the results from a linear regression analysis, none of the IVs can significantly influence the academic writing publication outcome (*p* > .05).

**Table 8:** Summary of linear regression analysis for variables predicting the academic writing publication outcome.

IV	*b*	SE	*t*	*p*
*Supervisor Support*	.321	0.625	.513	.609
*Year of study*	.613	0.374	1.638	.106
*Meeting frequency*	.148	0.229	.648	.520
*Learning Environment*	-.047	0.767	-.061	.951
*Skills Development*	-.279	0.620	-.450	.654
*Research Components*	-.259	0.534	-.486	.629
*Collaboration and Publication*	-.260	0.514	-.506	.615
*Teaching and Learning Quality*	-.161	0.706	-.228	.821
*Student Motivation*	.255	0.802	.317	.752
*Student self-efficacy*	-.585	0.436	-1.342	.185

### Results of factors affecting the academic writing self-efficacy

**Fig 2:**
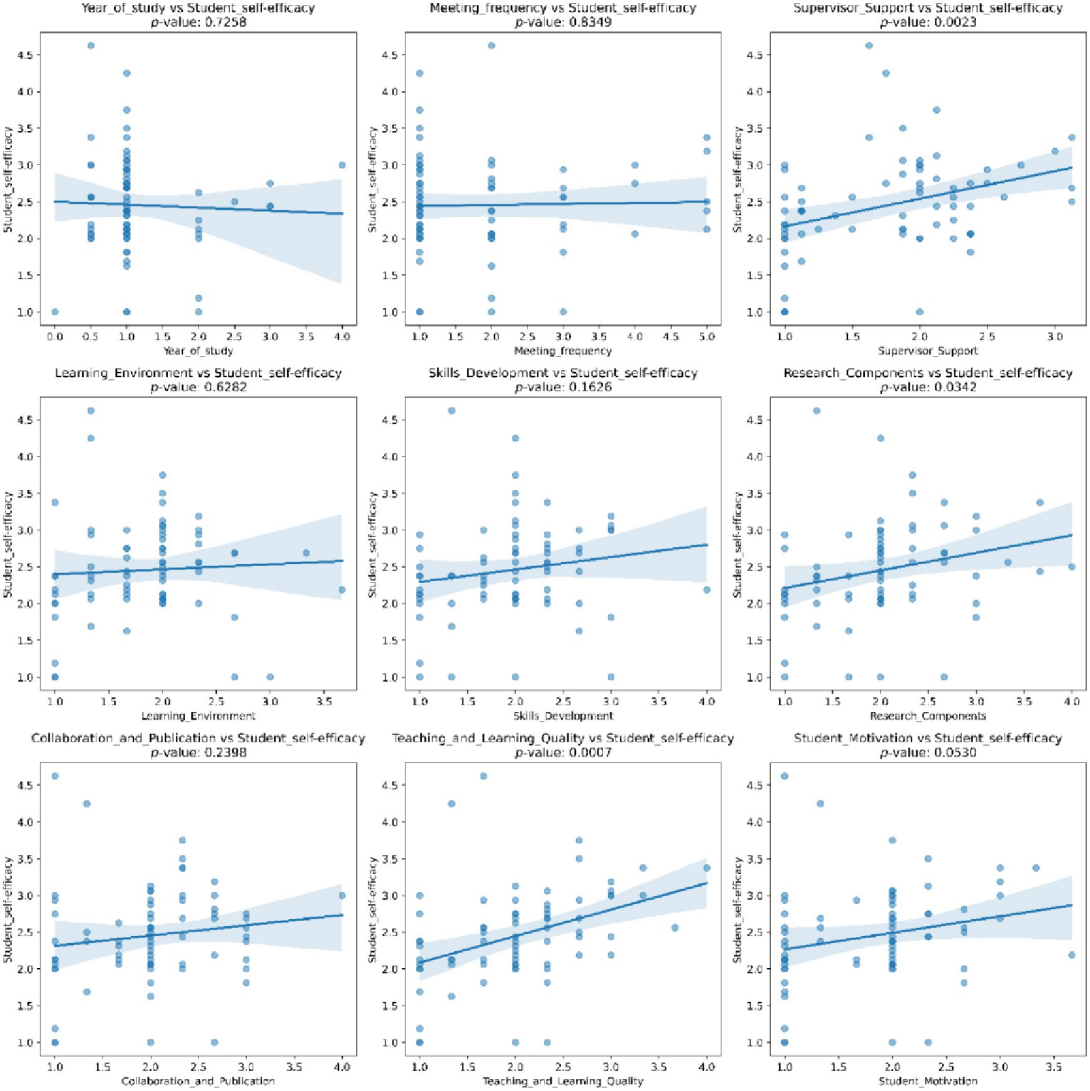
Regression plots illustrating relationships between academic and environmental factors and academic writing self-efficacy. This figure shows scatter plots examining the relationships between nine independent variables (e.g., supervisor support, learning environment, skill development, research component, teaching-learning quality, motivation, collaboration and publication, year of study, and meeting frequency) and the dependent variable, academic writing self-efficacy (scale: 1 to 4.5). Regression lines and 95% confidence intervals are included. Significant relationships (p < 0.05) were observed for supervisor support, research component, and teaching-learning quality, while other factors showed no significant correlation.

In this regression analysis, several IVs were above 5 [35]. To ensure reliable results, separate regressions were needed. The regression results and regression plots are displayed separately in Table 9 and Fig 2. SS (F = 9.966, *p* < .01), RC (F = 4.662, *p* < .05) and TL (F = 12.606, *p* < .01) were the most important predictors of SE. However, year of study, meeting frequency, LE, SD, MO, and CP (*p* > .05) do not significantly influence the academic writing self-efficacy.

**Table 9:** Summary of linear regression analysis for variables predicting the academic writing self-efficacy.

IV	*b*	SE	*t*	*p*
Supervisor Support	.376	.119	3.157	.002**
Year of study	-.041	.116	-.352	.726
Meeting frequency	.014	.066	.209	.835
Learning Environment	.068	.140	.486	.628
Skills Development	.169	.120	1.411	.163
Research Components	.240	.111	2.159	.034*
Collaboration and Publication	.140	.118	1.185	.240
Teaching and Learning Quality	.361	.102	3.550	.001**
Student Motivation	.225	.114	1.968	.053

*Note*:

*p< .05,

**p< .01

## Discussion

This study provides a focused analysis of the primary challenges faced by early-stage postgraduate students in academic writing, particularly in paper structure, language expression, and English language barriers. While literature search, research methodology selection, data analysis, and defining research topics were identified as significant obstacles, the findings emphasize a deeper issue: a lack of understanding of the fundamental components of academic papers, especially in writing the literature review section. These results align with Qasem and Zayid [11], yet this study adds a nuanced perspective by highlighting the distinct struggles in constructing specific sections of a paper. The challenges of clear and logical language expression, consistent with Jin et al. [12] and Latif et al. [3], further underscore the students’ emphasis on formality and academic rigor in language use, which directly impacts the quality and self-efficacy of their academic writing. Additionally, difficulties in using English as a second language resonate with findings from Qasem and Zayid [11], reaffirming the importance of English language proficiency in academic writing success.

To address these challenges, the study identifies multifaceted support requirements for early-stage postgraduates. Informational support—particularly academic writing guidance and mentorship—emerges as a critical need. These findings echo Jeyaraj et al. [13], who emphasized the necessity of assisting students with academic vocabulary, argument construction, and mentor feedback on language use. Beyond mentorship, students highlighted the importance of practical tools, such as data analysis software and literature management systems, as well as institution-level interventions, including writing courses and seminars. Interestingly, self-reflection and self-assessment were preferred over mentor feedback, reflecting Chung et al. [8], who noted the efficacy of self-evaluation in enhancing self-efficacy and writing outcomes.

This study also investigated factors influencing academic publication outcomes among early-stage postgraduate students. Contrary to expectations, year of study, meeting frequency, supervisor support (SS), and components of the teaching-research nexus (LE, SD, RC, TL, MO, CP) did not significantly affect publication success. This divergence from studies by Teng and Wang [17], Sun et al. [16], and Samanhudi and Linse [18] may be attributed to the nascent stage of the participants’ academic journeys. Early-stage students often lack sufficient academic writing experience and are primarily engaged in mandatory coursework, limiting their ability to publish. Reflective journals corroborated these findings, revealing a fundamental lack of understanding of the publication process. These insights underscore the importance of tailored support for early-stage students to address these specific challenges.

Conversely, the analysis identified supervisor support (SS) as a significant predictor of academic writing self-efficacy. Guidance from instructors not only improves students’ writing confidence but also equips them with practical skills in planning, drafting, and revising academic texts, as supported by Chung et al. [8]. Within the teaching-research nexus, research component (RC) also emerged as a key factor influencing self-efficacy. RC encapsulates critical elements such as problem identification, literature review, methodology, and discussion, providing students with a structured framework for academic writing. Strengthening students’ understanding of these elements is likely to bolster their confidence, suggesting that targeted interventions in these areas can be transformative. Furthermore, teaching-learning quality (TL) positively impacts self-efficacy by enriching students’ understanding of their subject area and exposing them to innovative research practices. Similar findings by Jeyaraj et al. [13] reinforce the value of integrating research-based teaching methods into postgraduate curricula to motivate students and enhance their academic self-confidence.

Notably, factors such as year of study, meeting frequency, learning environment (LE), skill development (SD), motivation (MO), and collaboration/publication (CP) did not significantly predict academic writing self-efficacy. This discrepancy from earlier studies [9, 24] may stem from the unique context of this research, focusing on early-stage postgraduate students who face distinct challenges compared to their more advanced peers. Additionally, cultural and institutional factors within Chinese higher education may shape these outcomes differently. Unlike prior research that examined the effects of self-efficacy on learning motivation (e.g., Schunk and DiBenedetto [25]), this study innovatively explored the predictors of self-efficacy itself. These differences highlight the importance of contextual considerations when interpreting academic writing outcomes.

## Conclusion and future research

This study thoroughly examined the challenges and support needs of early-stage postgraduate students in academic writing. Through thematic analysis of reflective diaries, several significant findings emerged. The primary challenges include mastering the basic framework of papers, such as literature review retrieval and writing, selecting research methods, analyzing and collecting data, and choosing research topics. Additionally, students face challenges with academic language expression and proficiency in reading and writing in English.

The support needed by early-stage postgraduate students focuses on informational support, including guidance from faculty and access to knowledge resources; instrumental support, such as tools and services; and support for self-reflection and self-assessment. Furthermore, this study explored factors affecting academic publication outcomes and academic self-efficacy, focusing on the year of study, meeting frequency, supervisor support (SS), and the teaching-research nexus (learning environment (LE), skill development (SD), research component (RC), teaching and learning (TL), motivation (MO), collaboration and publication (CP)). Linear regression analysis indicated that while the year of study, meeting frequency, and the teaching-research nexus did not significantly impact academic publication outcomes, separate regression analysis indicated that SS, RC, and TL significantly influenced students’ self-efficacy.

These findings highlight the deficiencies of early-stage postgraduate students in foundational writing skills and underscore the complexity of factors affecting academic publication outcomes and self-efficacy. Higher education institutions and educators must continue to explore and implement research-driven teaching, providing timely guidance and feedback for students’ academic writing. This approach is essential for creating a learning environment that fosters growth and develops the confidence necessary for academic success.

However, the study is not without its limitations. Firstly, the research is constrained by a relatively small sample size, which may restrict the generalizability of the findings. Future research should aim to expand the sample size to enhance the generalizability and external validity of the findings. Employing stratified or random sampling methods could ensure a more representative sample of postgraduate students across various disciplines and institutions. Additionally, future studies should include postgraduate students at different stages of their academic journey, facilitating comparative analyses on the factors influencing academic publication outcomes and writing self-efficacy at different educational levels. Investigating these aspects will provide a comprehensive understanding of the dynamics of academic writing and publication outcomes, enabling the development of more tailored and effective support mechanisms for students throughout their academic careers. This broader approach will inform strategies to better support postgraduate students in overcoming writing challenges and achieving academic success.

## Supporting information

S1 Appendix Research questionnaire. This questionnaire consists of three sections: The Teaching-Research Nexus Questionnaire, The Postgraduate Research Outcomes Questionnaire, and The Situated Academic Writing Self-Efficacy Scale (SAWSES), covering items related to these areas.
(DOCX)

## References

[1] Khalaf M. A., Alshammari A. Effects of project-based learning on postgraduate students’ research proposal writing skills. European Journal of Educational Research. 2023; 12(1): 189–200. doi: 10.12973/eu-jer.12.1.189

[2] Pınar Y., D. Gür N. K. Pınar K. Demir E. K. Iltar S. A. et al. A comparative study of postgraduate theses in pedagogy and preschool education in Austria and Turkey. Frontiers in Psychology. 2023; 13: 1051923. doi: 10.3389/fpsyg.2022.1051923 36687857 PMC9853051

[3] Latif F., Nijabat A., Sohail M., Sohail E. ACADEMIC WRITING CHALLENGES: PERSPECTIVES OF MASTERS AND DOCTORAL STUDENTS. Pakistan Journal of Society, Education and Language. 2023; 9(2): 205–212. https://www.researchgate.net/publication/360937789

[4] Lee J. C. K. Teachers’ work, change and learning: roles, contexts and engagement. Teachers and Teaching. 2019; 25(4): 399–403. doi: 10.1080/13540602.2019.1625616

[5] Hu S., Li Y., Zheng J. Integrating research and teaching in Chinese universities: Perceptions and practices. Journal of Higher Education Policy and Management. 2019; 41(5): 519–536. doi: 10.1080/1360080X.2019.1634672

[6] Li M., Zheng Y. Research-teaching nexus in Chinese higher education: A qualitative study. Higher Education Research and Development. 2018; 37(4): 757–771. doi: 10.1080/07294360.2018.1446417

[7] Bandura A. Self-efficacy: The exercise of control. New York: Freeman; 1997.

[8] Chung H. Q., Chen V., Olson C. B. The impact of self-assessment, planning and goal setting, and reflection before and after revision on student self-efficacy and writing performance. Reading and Writing. 2021; 34(7): 1885–1913.

[9] Vincent C., Tremblay-Wragg É., Déri C., Plante I., Mathieu C. S. How writing retreats represent an ideal opportunity to enhance PhD candidates’ writing self-efficacy and self-regulation. Teaching in Higher Education. 2023; 28(7):1600–1619.

[10] Rahimi M., Fathi J. Exploring the impact of wiki-mediated collaborative writing on EFL students’ writing performance, writing self-regulation, and writing self-efficacy: a mixed methods study. Computer Assisted Language Learning. 2022; 35(9): 2627–2674.

[11] Qasem F. A. A., Zayid E. I. M. 2019. The challenges and problems faced by students in the early stage of writing research projects in L2, University of Bisha, Saudi Arabia. European Journal of Special Education Research. 4(1).

[12] Jin X., Jiang Q., Xiong W., Feng Y., Zhao W. Effects of student engagement in peer feedback on writing performance in higher education. Interactive Learning Environments. 2024; 32(1):128–143. doi: 10.1080/10494820.2022.2081209

[13] Joanna Joseph Jeyaraj. It’s A Jungle Out There: Challenges In Postgraduate Research Writing. Journal of Language Studies. 2018; 18(1): 22–33. doi: 10.17576/gema-2018-1801-02

[14] Hawari O. M. A., Al-Shboul Y., Huwari I. F. Supervisors’ perspectives onpostgraduate students’ problems in academic writing. European Journal of Educational Research. 2022; 11(1):545–556. doi: 10.12973/eu-jer.11.1.545

[15] Shen C., Guo J., Shi P., Qu S., Tian J. A corpus-based comparison of syntactic complexity in academic writing of L1 and L2 English students across years and disciplines. Plos one. 2023; 18(10): e0292688. doi: 10.1371/journal.pone.0292688 37812624 PMC10561866

[16] Sun T., Wang C., Lambert R. G., Liu L. Relationship between second language English writing self-efficacy and achievement: A meta-regression analysis. Journal of Second Language Writing. 2021; 53:100817. doi: 10.1016/j.jslw.2021.100817

[17] Teng M. F., Wang C. Assessing academic writing self‐efficacy belief and writing performance in a foreign language context. Foreign Language Annals. 2023; 56(1): 144–169. doi: 10.1111/flan.12638

[18] Samanhudi U., Linse C. Critical thinking-related challenges to academic writing: A case of Indonesian postgraduate students at a UK University. Lingua Cultura. 2019; 13(2): 107–114. doi: 10.21512/lc.v13i1.5122

[19] Aydın G., Baysan S. Perceptions of postgraduate students on academic writing skills: A metaphor analysis study. Journal of Language and Linguistic Studies. 2018; 14(2): 212–239.

[20] Casanave C. P., Hubbard P. The writing assignments and writing problems of doctoral students: Faculty perceptions, pedagogical issues, and needed research. English for Specific Purposes. 1992; 11(1): 33–49.

[21] Lin L. H. F., Morrison B. Challenges in academic writing: Perspectives of Engineering faculty and L2 postgraduate research students. English for Specific Purposes. 2021; 63: 59–70. doi: 10.1016/j.esp.2021.03.004

[22] Wischgoll A. Improving undergraduates’ and postgraduates’ academic writing skills with strategy training and feedback. Frontiers in Education. 2017; 2:33.

[23] Pajares F., Valiante G., Cheong Y. F. Writing self-efficacy and its relation to gender, writing motivation and writing competence: A developmental perspective. Writing and motivation. Brill; 2006. pp. 141–159.

[24] Izawa Y., FUJIMORI C., GODFREY C., and OIDA Y. Relationships among self-efficacy, willingness, and writing performance in an academic writing program. Fora. 2017; (1): 12–22.

[25] Schunk D. H., DiBenedetto M. K. Self-efficacy and human motivation. In Advances in motivation science. Elsevier; 2021. pp. 153–179.

[26] House J., Kahn R. Measures and concepts of social support. In: Cohen S. and Syme S. (Eds) Social Support and Health. Academic Press; 1985.

[27] Yang B. Academic Writing Coursebook for Postgraduates. Fudan University Press; 2021.

[28] Biernacki P., Waldorf D. Snowball sampling: Problems and techniques of chain referral sampling. Sociological Methods and Research. 1981; 10(2): 141–163.

[29] Goodman L.A. Snowball sampling. Annals of Mathematical Statistics. 1961; 32: 148–170.

[30] Atkinson R., Flint J. Accessing hidden and hard-to-reach populations: Snowball research strategies. Social Research Update. 2001; 33: 1–4.

[31] Creswell J. W., Clark V. L. P., Gutmann M. L., Hanson W. E. Advanced mixed. Handbook of mixed methods in social & behavioral research. 2003; 209: 209–240.

[32] Devesh S., Nanjundaswamy A. Cultivating a culture of inquiry: Exploring the factors influencing the integration of research and teaching in higher education institutions. Journal of Applied Research in Higher Education. 2023. doi: 10.1108/JARHE-06-2023-0227

[33] Mitchell K. M., McMillan D. E., Lobchuk M. M., Nickel N. C., Rabbani R., et al. Development and validation of the situated academic writing self-efficacy scale (SAWSES). Assessing Writing. 2021; 48: 100524. doi: 10.1016/j.asw.2021.100524

[34] Braun V., Clarke V. Using thematic analysis in psychology. Qualitative research in psychology. 2006; 3(2): 77–101.

[35] Menard S. Applied logistic regression analysis. Sage; 2002.

