# Supplementary material for: Academic writing challenges and supports for early-stage Chinese postgraduates: A mixed-methods study on teaching-research integration, supervisor engagement, and self-efficacy
Source: PLoS One. 2025 Feb 14;20(2):e0317470. doi: 10.1371/journal.pone.0317470 (PMC11828370; doi:10.1371/journal.pone.0317470)
Supplement: S1 Appendix — This questionnaire consists of three sections: The Teaching-Research Nexus Questionnaire, The Postgraduate Research Outcomes Questionnaire, and The Situated Academic Writing Self-Efficacy Scale (SAWSES), covering items related to these areas. (DOCX) [file pone.0317470.s001.docx]

**Appendix A：**

1.Nick name:

2.Type of the University:

A. Project 985 University B. Project 211 University C. Double First Class University D. Common Higher Education Institutions (Including universities and institutions)

3. Grade

A Year 1 B Year 2 C Year 3 D Year 4

4. Major: ____________

5. Gender:

A Male B Female

6. How often do you meet with your supervisor to discuss your research project?"

A. Weekly B. Bi-weekly C. Monthly D. Less than monthly E. Not applicable

7. The guidance from my supervisor significantly contributes to the progress of my research project.

A. Totally Agree B. Agree C. Neither Agree nor Disagree D. Disagree E. Totally Disagree

8. My supervisor is easily accessible when I need advice or feedback.

A. Totally Agree B. Agree C. Neither Agree nor Disagree D. Disagree E. Totally Disagree

9. The feedback from my supervisor helps me improve my research skills and project quality.

A. Totally Agree B. Agree C. Neither Agree nor Disagree D. Disagree E. Totally Disagree

10. My supervisor encourages me to develop and pursue my research ideas.

A. Totally Agree B. Agree C. Neither Agree nor Disagree D. Disagree E. Totally Disagree

11. My supervisor provides support and encouragement that helps me overcome academic and emotional challenges,

A. Totally Agree B. Agree C. Neither Agree nor Disagree D. Disagree E. Totally Disagree

12. My supervisor actively supports my professional development and networking within my field.

A. Totally Agree B. Agree C. Neither Agree nor Disagree D. Disagree E. Totally Disagree

13. I receive timely and actionable feedback from my supervisor on my research work

A. Totally Agree B. Agree C. Neither Agree nor Disagree D. Disagree E. Totally Disagree

14. My supervisor’s research interests align well with my own, enhancing the research experience.

A. Totally Agree B. Agree C. Neither Agree nor Disagree D. Disagree E. Totally Disagree

15. My teachers discuss their as well as others research findings in the class.

A. Totally Agree B. Agree C. Neither Agree nor Disagree D. Disagree E. Totally Disagree

16. I became familiar with reading and understanding the results of scientific research.

A. Totally Agree B. Agree C. Neither Agree nor Disagree D. Disagree E. Totally Disagree

17. My University provides an opportunity to participate/attend research seminars, symposiums, and conferences in or outside the University.

A. Totally Agree B. Agree C. Neither Agree nor Disagree D. Disagree E. Totally Disagree

18. The case studies were included as a part of our course curriculum.

A. Totally Agree B. Agree C. Neither Agree nor Disagree D. Disagree E. Totally Disagree

19. I have used previous research papers as a reference as part of my studies.

A. Totally Agree B. Agree C. Neither Agree nor Disagree D. Disagree E. Totally Disagree

20. The “research methodology” course was taught as a subject in a degree programme.

A. Totally Agree B. Agree C. Neither Agree nor Disagree D. Disagree E. Totally Disagree

21. University conducts workshops/ special lectures about how to write assignments and research papers.

A. Totally Agree B. Agree C. Neither Agree nor Disagree D. Disagree E. Totally Disagree

22. University guides to develop my research skills.

A. Totally Agree B. Agree C. Neither Agree nor Disagree D. Disagree E. Totally Disagree

23. My knowledge of important aspects of conducting research has increased.

A. Totally Agree B. Agree C. Neither Agree nor Disagree D. Disagree E. Totally Disagree

24. My course-related assignments involve the collection and analysis of primary or secondary data.

A. Totally Agree B. Agree C. Neither Agree nor Disagree D. Disagree E. Totally Disagree

25. I have to undertake a research project as part of my degree requirement.

A. Totally Agree B. Agree C. Neither Agree nor Disagree D. Disagree E. Totally Disagree

26. University encourages and supports students to present papers or research posters at research conferences in/outside the University.

A. Totally Agree B. Agree C. Neither Agree nor Disagree D. Disagree E. Totally Disagree

27. My teachers arrange group discussions and tutorials for critical evaluation of my assignments.

A. Totally Agree B. Agree C. Neither Agree nor Disagree D. Disagree E. Totally Disagree

28. The University provides opportunities to participate in student research paper competition in/outside

University.

A. Totally Agree B. Agree C. Neither Agree nor Disagree D. Disagree E. Totally Disagree

29. My teachers encourage the publication of research papers in journals, books, and magazines jointly with the students.

A. Totally Agree B. Agree C. Neither Agree nor Disagree D. Disagree E. Totally Disagree

Teaching and Learning Quality -QTR

30. The research and inquiry-based assignments/research projects have enhanced my learning process.

A. Totally Agree B. Agree C. Neither Agree nor Disagree D. Disagree E. Totally Disagree

31. I can integrate theory and practical aspects of the courses/subject to solve real-life problems.

A. Totally Agree B. Agree C. Neither Agree nor Disagree D. Disagree E. Totally Disagree

32. The research-based activities of the courses/subjects have enhanced my employability skills.

A. Totally Agree B. Agree C. Neither Agree nor Disagree D. Disagree E. Totally Disagree

33. I was inspired to learn more about my field of study.

A. Totally Agree B. Agree C. Neither Agree nor Disagree D. Disagree E. Totally Disagree

34. I have developed more interest in doing further research in the field of my study.

A. Totally Agree B. Agree C. Neither Agree nor Disagree D. Disagree E. Totally Disagree

35. My interest in learning has enhanced due to the integration of teaching with research.

A. Totally Agree B. Agree C. Neither Agree nor Disagree D. Disagree E. Totally Disagree

36.Even when the writing is hard, I can find ways to overcome my writing difficulties.

A. Totally Agree B. Agree C. Neither Agree nor Disagree D. Disagree E. Totally Disagree

37. I can successfully use scholarly academic words and phrases when writing in my courses. (courses/program for grad student survey)

A. Totally Agree B. Agree C. Neither Agree nor Disagree D. Disagree E. Totally Disagree

38.I can combine or synthesize multiple sources I’ve read to create an original product or text.

A. Totally Agree B. Agree C. Neither Agree nor Disagree D. Disagree E. Totally Disagree

39.When I write, I can think about my audience and write so they clearly understand my meaning.

A. Totally Agree B. Agree C. Neither Agree nor Disagree D. Disagree E. Totally Disagree

40.When I receive feedback on my writing, no matter how it makes me feel, I can use that feedback to improve my writing in the future.

A. Totally Agree B. Agree C. Neither Agree nor Disagree D. Disagree E. Totally Disagree

41.When I reflect on what I am writing I can make my writing better.

A. Totally Agree B. Agree C. Neither Agree nor Disagree D. Disagree E. Totally Disagree

42.When I read articles about my topic, the connections I feel with the ideas of other authors can inspire me to express my own ideas in writing.

A. Totally Agree B. Agree C. Neither Agree nor Disagree D. Disagree E. Totally Disagree

43.When I look at the overall picture I’ve presented in my writing, I can assess how all the pieces tell the complete story of my topic or argument.

A. Totally Agree B. Agree C. Neither Agree nor Disagree D. Disagree E. Totally Disagree

44.I can recognize when I’ve wandered away from writing what my audience needs to know and have begun writing about interesting, but unrelated, ideas.

A. Totally Agree B. Agree C. Neither Agree nor Disagree D. Disagree E. Totally Disagree

45.With each new writing assignment, I can adapt my writing to meet the needs of that assignment.

A. Totally Agree B. Agree C. Neither Agree nor Disagree D. Disagree E. Totally Disagree

46.When I seek feedback on my writing, I can decide when that feedback should be ignored or incorporated into a revision in my writing.

Totally Agree B. Agree C. Neither Agree nor Disagree D. Disagree E. Totally Disagree

47.I can use creativity when writing an academic paper.

A. Totally Agree B. Agree C. Neither Agree nor Disagree D. Disagree E. Totally Disagree

48.I feel I can give my writing a creative spark and still sound professional.

A. Totally Agree B. Agree C. Neither Agree nor Disagree D. Disagree E. Totally Disagree

49.I feel I can develop my own writing voice (ways of speaking in my writing that are uniquely me).

A. Totally Agree B. Agree C. Neither Agree nor Disagree D. Disagree E. Totally Disagree

50.Even with very specific assignment guidelines, I can find ways of writing my assignment to make it original or unique.

A. Totally Agree B. Agree C. Neither Agree nor Disagree D. Disagree E. Totally Disagree

51.I can comfortably express the concepts, language, and values of my discipline or major in my writing assignments

A. Totally Agree B. Agree C. Neither Agree nor Disagree D. Disagree E. Totally Disagree

52. Please write the numbers of Peer-Reviewed Journal Publications (Past 3 Years):

SCI/SSCI/AandHCI indexed: ______

ESCI/Scopus indexed: ______

International (non-indexed): ______

CSSCI or CSSCI (Expanded): ______

National/Local Chinese journals: ______

Total Citations Received (Past 5 Years): ______

1. Completed Research Projects (Past 3 Years):

(Please list each project with a brief description, your role, and project level.)

1. Please list any received Awards and Honors (Past 3 Years):
2. How many times have you been invited to speak at conferences or academic workshops? (Past 3 Years):
3. How many patents resulted from your research? (Past 3 Years):
4. Include any other research outcomes or impacts not covered above:
